# Disseminated Varicella-Zoster Virus in a Patient on Pegloticase and Mycophenolate Mofetil for Gout

**DOI:** 10.7759/cureus.81921

**Published:** 2025-04-08

**Authors:** Sylvia Riad, Kimberly D Johnson

**Affiliations:** 1 Internal Medicine, Methodist Dallas Medical Center, Dallas, USA

**Keywords:** gout, immunosuppression, mycophenolate mofetil, secondary immunodeficiency, varicella-zoster virus

## Abstract

There are various treatments for managing gout, including combination treatment with immunomodulators. However, these treatments can lead to significant immunosuppression, potentially leading to worse health outcomes, such as the one described in this case.

We present the case of a 70-year-old female patient with a past medical history of atrial fibrillation, type 2 diabetes mellitus, and gout, who presented with progressively worsening altered mental status. This ultimately necessitated intubation, as well as the initiation of vasopressor support for new, worsening hypotension. She was noted to have a widespread vesicular pruritic rash present for the past month. Swabs of the vesicular lesions resulted positive for varicella-zoster virus (VZV). Her serum was also VZV positive, with 6,800,000 copies/mL detected. She later developed bilateral patchy infiltrates, and a bronchoscopy showed patchy erythema in multiple proximal airways. A lumbar puncture was performed due to the patient's altered mental status, which showed VZV (<200 copies) in her cerebrospinal fluid (CSF). She was, therefore, started on acyclovir, and she clinically improved. Her final diagnosis was disseminated VZV with multiorgan involvement, including the lung, skin, liver, and CSF. Medication review revealed that the patient was taking mycophenolate mofetil (MMF) and pegloticase for the treatment of gout. It was thus suspected that the MMF led to an immunocompromised state, which predisposed her to disseminated VZV.

Pegloticase is a Food and Drug Administration (FDA)-approved treatment for refractory gout but is known to be highly immunogenic. To reduce pegloticase's immunogenicity, MMF is often co-administered. In this case, our patient became profoundly immunosuppressed with the MMF-pegloticase regimen, which led to disseminated VZV. This case sheds light on the serious risks associated with this drug regimen.

## Introduction

Gout is a common form of inflammatory arthritis that occurs when uric acid crystals accumulate in the joints, leading to sudden and severe pain, redness, and swelling. It often affects the lower limb, classically the first metatarsophalangeal joint, but can also impact other joints, including ankles, knees, wrists, elbows, and hand joints [1]. Gout typically develops due to urate overproduction or underexcretion, causing the formation of monosodium urate crystals [2]. Risk factors for gout include obesity, hypertension, excessive alcohol consumption, a diet rich in purines (such as red meat and seafood), and certain medications [1]. Multiple treatment modalities have been described for the treatment of gout. Recent advances in gout treatment have introduced several new therapies to improve management and reduce the frequency and severity of gout attacks. These include combination treatment with immunomodulators. However, these treatments can lead to significant immunosuppression, potentially leading to worse health outcomes. 

Here, we describe a patient with gout who developed severe side effects due to treatment for the condition.

This article was previously accepted as a meeting abstract at the 2025 American Academy of Allergy, Asthma, and Immunology/World Allergy Organization Joint Congress and was presented on February 28, 2025.

## Case presentation

A 70-year-old female patient with a past medical history of atrial fibrillation, non-ischemic cardiomyopathy (status post dual-chamber implantable cardioverter defibrillator placement), chronic kidney disease, type 2 diabetes mellitus, and gout presented to the emergency department (ED) with a one-week history of abdominal pain.

Prior to admission, the patient had presented to the ED twice with sharp, stabbing upper abdominal pain, myalgia, nausea, and a rash. She reported first noticing the rash around her buttocks, which subsequently spread to involve her whole body, including her hands and face. The rash was described as itchy and painful erythematous papules. She denied any recent travel or exposure to new soaps, detergents, or foods and reported no known ill contacts.

**Figure 1:**
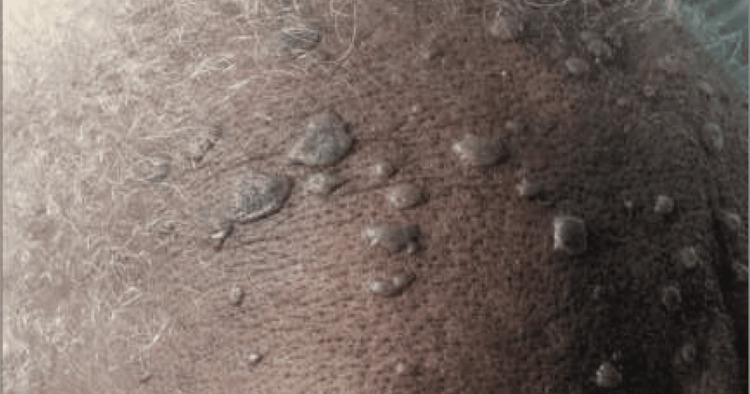
Images of the vesicular rash on the patient's forehead

**Figure 2:**
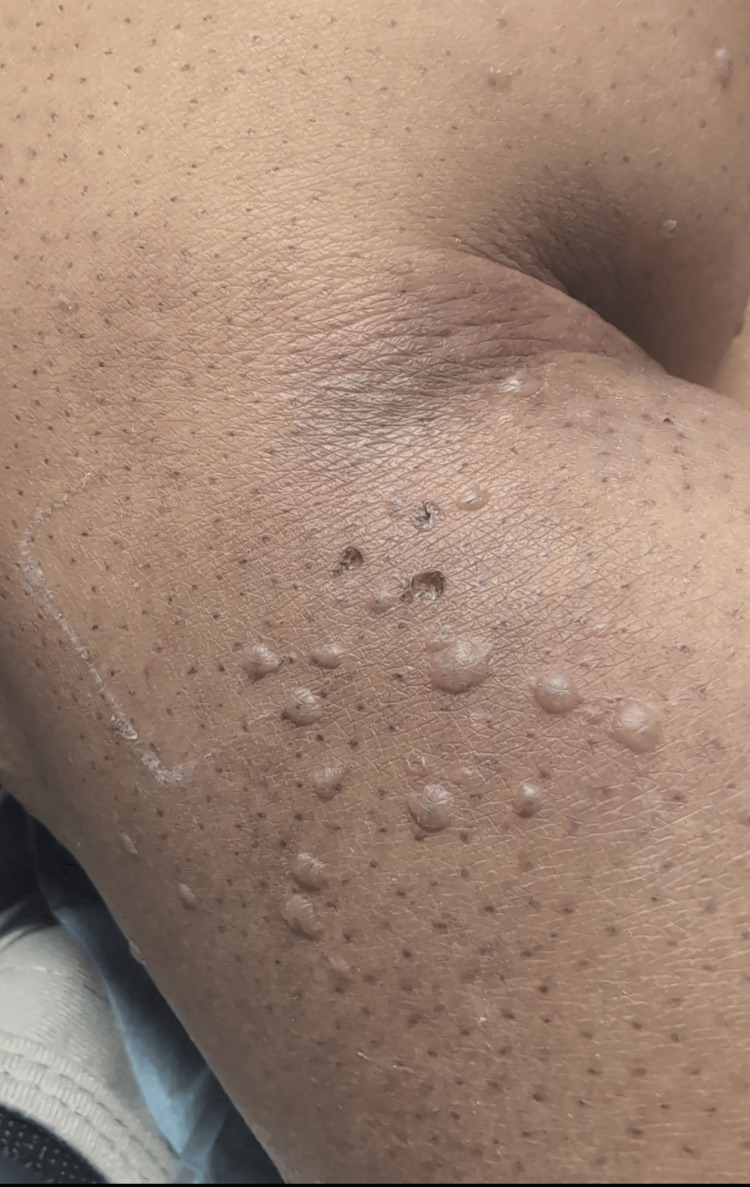
Images of the vesicular rash on the patient's right arm

Physical examination revealed a diffuse vesicular rash, heavily concentrated on the lower back, with additional rash noted on the face, particularly the forehead, as shown in Figure 1, bilateral arms, legs, and neck. Images of the vesicular rash on the right arm are demonstrated in Figure 2.

**Figure 3:**
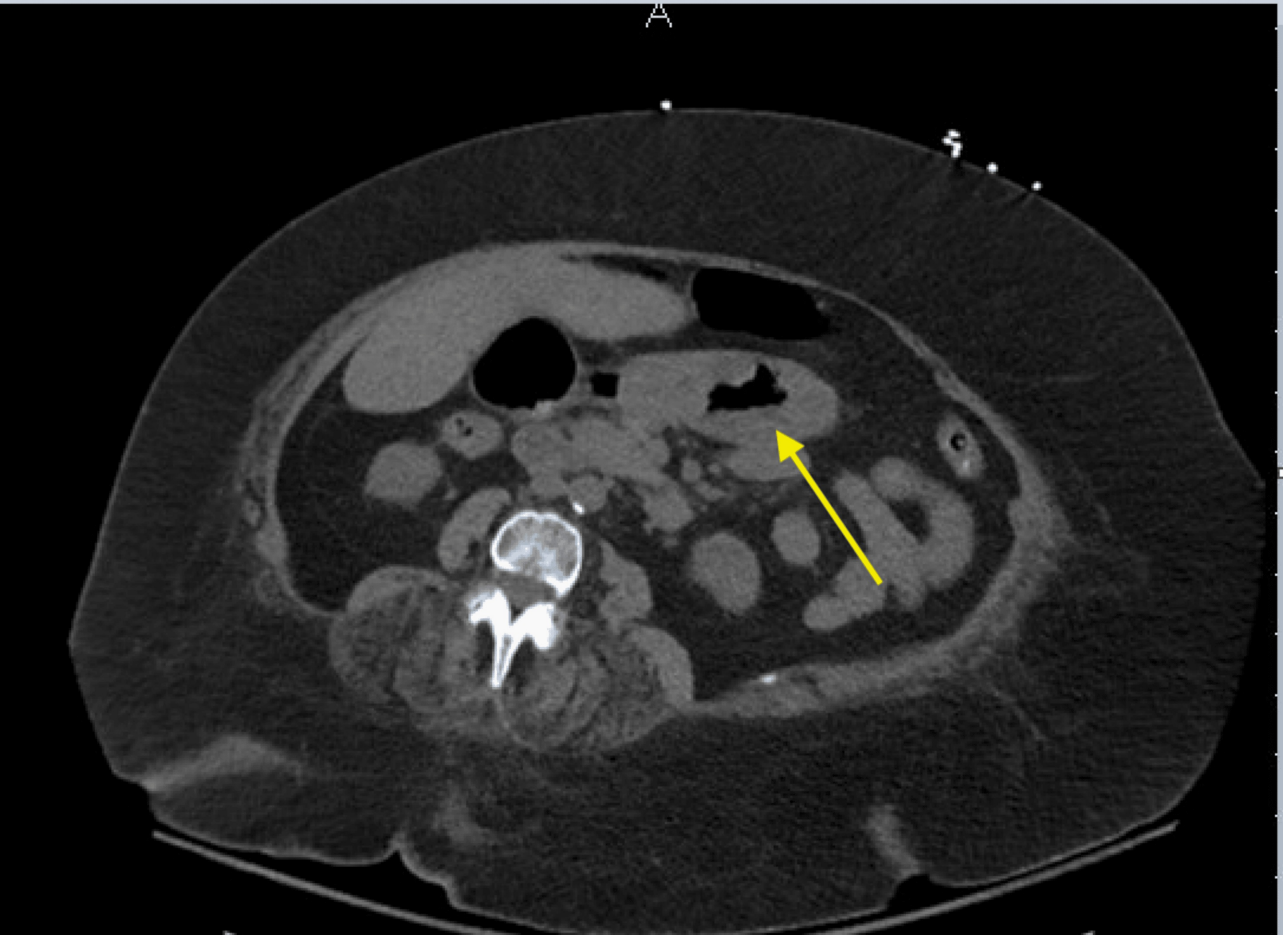
Computed tomography image of the abdomen/pelvis without contrast showing moderate gastric wall thickening (arrow)

There was no lip or tongue swelling or oral mucosal lesions. Based on the appearance and distribution of the rash, the ED physician suspected dermatitis herpetiformis. A computed tomography scan of the abdomen and pelvis was obtained to evaluate her abdominal pain, which revealed moderate subjective thickening of the gastric body and antrum wall, which was concerning for gastritis, as shown in Figure 3. 

Laboratory results, as shown in Table 1, were significant for creatinine of 1.47 mg/dL, which was improved compared to baseline, but her liver enzymes were elevated: aspartate aminotransferase (AST) of 271 U/L, alanine transaminase (ALT) of 193 U/L, alkaline phosphatase (ALP) of 137 U/L, and total bilirubin of 0.5 mg/dL. The ED physician suspected celiac disease, given the findings of gastritis and the rash. The patient was discharged from the emergency room, with instructions to follow up with her primary care physician.

**Table 1:** Laboratory values on initial presentation to the emergency room AST: aspartate aminotransferase; ALT: alanine aminotransferase; ALP: alkaline phosphatase

Laboratory test	Result	Units	Normal range
Creatinine	1.47	mg/dL	0.70-1.40 mg/dL
AST	271	U/L	8-42 U/L
ALT	193	U/L	<35 U/L
ALP	137	U/L	38-126 U/L
Total bilirubin	0.5	mg/dL	0.0-1.4 mg/dL

The next day, the patient returned to the ED with a complaint of altered mental status and lethargy. She was febrile (temperature: 38.8°C), tachycardic (heart rate: 110 bpm), tachypneic (respiratory rate: 40 breaths per minute), and hypotensive. Her husband reported that she was having abdominal pain, nausea, progressive lethargy, and a vesicular rash ongoing for a month. Laboratory findings, as demonstrated in Table 2, were significant for a white blood cell (WBC) count of 5.3×10^3^/uL, blood glucose level of 584 mg/dL, bicarbonate of 10 mmol/L, anion gap of 17 mEq/L, creatinine of 2.43 mg/dL, beta-hydroxybutyrate of 3.99 mmol/L, AST of 625 U/L, ALT of 365 U/L, and procalcitonin of 6.87 ng/mL. A diagnosis of diabetic ketoacidosis (DKA) with concurrent sepsis was made. The DKA protocol was initiated, and antibiotics (vancomycin and ceftriaxone) were started. The patient continued to be tachycardic and hypotensive, requiring norepinephrine initiation. She continued to be tachypneic with worsening mentation, subsequently requiring intubation. Worsening shock required the addition of vasopressin. The infectious disease team was consulted to assist in managing the vesicular lesions, and the nephrology team was consulted for the management of worsening acute kidney injury, as indicated by an increased creatinine level of 3.3 mg/dL. Swabs of the vesicular lesions were obtained to rule out monkeypox, varicella-zoster virus (VZV), and herpes simplex virus (HSV). A bronchoscopy was performed, given the presence of bilateral patchy infiltrates on the chest X-ray and the patient's worsening hypoxia.

**Table 2:** Laboratory values upon admission to the hospital AST: aspartate aminotransferase; ALT: alanine aminotransferase

Laboratory test	Result	Units	Normal range
White blood cell count	5.3×10³	/µL	3.8-11.0×10³/µL
Blood glucose level	584	mg/dL	70-110 mg/dL
Bicarbonate	10	mmol/L	22-31 mmol/L
Anion gap	17	mEq/L	8-16 mEq/L
Creatinine	2.43	mg/dL	0.70-1.40 mg/dL
Beta-hydroxybutyrate	3.99	mmol/L	0.02-0.27 mmol/L
AST	625	U/L	8-42 U/L
ALT	365	U/L	<35 U/L
Procalcitonin	6.87	ng/mL	<0.5 ng/mL

Empiric acyclovir was initiated, and antibiotics were de-escalated to ceftriaxone and azithromycin to treat possible community-acquired pneumonia. Bronchoscopy showed patchy erythema in multiple proximal airways, including the distal right mainstem/right intermediate bronchus, left mainstem, and subsegmental bronchi. The serum VZV test was positive, with a viral load of 6,800,000 copies/mL. Concurrently, the lesion swab tested positive for VZV, and the tissue examination showed HSV cytopathic changes, which can be seen with VZV or HSV-1 and HSV-2. Bronchoscopy studies showed an elevated WBC count of 906 per cubic millimeter, with a polymorphonuclear leukocyte percentage of 64%. Cytomegalovirus (CMV) PCR of the tissue obtained from the bronchoscopy was positive, with a viral load of only 10,222 copies/mL detected. However, the infectious disease team had a lower suspicion that CMV was the primary pathogen, as it can be a colonizer in the setting of polymicrobial infections. The patient was diagnosed with disseminated VZV involving the skin, lungs, and liver, and acyclovir therapy was continued.

The patient's hospital course was complicated by worsening agitation and mentation, so lumbar puncture was pursued to rule out central nervous system viral involvement. While the cerebrospinal fluid (CSF) culture had few WBCs and no organisms, it was positive for VZV with a viral load of <200 copies/mL, confirming a diagnosis of disseminated VZV with multiorgan involvement. The patient finished a 14-day course of acyclovir, during which her mentation gradually improved, and she was weaned off pressor support. A review of her medications revealed she had been on mycophenolate mofetil (MMF) in conjunction with pegloticase for the treatment of gout. It was suspected that the MMF led to an immunocompromised state, which caused the disseminated VZV.

## Discussion

Gout is a common disease that leads to many ED visits and is often a frequent topic of discussion with primary care providers and rheumatologists during office visits. Recent reports have estimated a global prevalence ranging from less than 1% to 6.8% and an incidence rate of 0.58-2.89 cases per 1,000 person-years [3]. Gout occurs due to the deposition of monosodium urate crystals in the joints, which triggers an inflammatory response. The crystals are phagocytosed by macrophages within the joint space, which triggers the formation of the NLRP3 inflammasome [4]. This activates caspase-1, an enzyme that converts pro-IL-1β to IL-1β, which triggers the production of various other pro-inflammatory cytokines. IL-1β promotes vasodilation, the recruitment of neutrophils, and the production of additional pro-inflammatory cytokines, perpetuating the inflammation in gouty arthritis [5].

Gout causes a significant economic burden, with an estimated $1 billion spent annually on outpatient treatments in the United States [6]. Many gout treatments aim to reduce symptoms during acute flares and prevent recurrences. Common treatments for acute flares include nonsteroidal anti-inflammatory drugs (NSAIDs), colchicine, steroids, and anti-IL-1β biologic therapies, although the latter has not been approved by the United States (US) Food and Drug Administration (FDA). For patients with recurrent gout attacks, rheumatologic guidelines recommend initiating urate-lowering therapies (ULT), such as xanthine oxidase inhibitors, uricosuric agents, and uricases such as pegloticase [7]. According to rheumatologic guidelines, ULT should be initiated in patients with one or more subcutaneous tophi, radiographic evidence of joint damage due to gout, or frequent flare-ups, defined as two or more attacks per year [8]. On the other hand, the American College of Physicians (ACP) advises against starting ULT in patients after their first attack or those with infrequent attacks (fewer than two per year). The ACP also highlights the importance of shared decision-making, urging healthcare providers to discuss the benefits and risks of ULT with patients before beginning treatment [6]. This discordance was reviewed by an expert panel convened by the Gout, Hyperuricemia, and Crystal-Associated Disease Network (G-CAN) [9]. 

Pegloticase, an FDA-approved medication for refractory gout, is a recombinant PEGylated uricase derived from non-human mammalian genes. It's one of the first biological treatments indicated for refractory gout [10]. Although FDA-approved, pegloticase isn't the first line of therapy for gout. In fact, the American College of Rheumatology strongly recommends against using pegloticase as the first line of therapy for gout [8]. Its use is also limited by immunogenicity. PEGylation reduces its immunogenicity; however, it does not eliminate it entirely. Anti-drug antibodies (ADA) develop in >40% of patients, potentially neutralizing the drug [7]. In a phase 3 study, ADA formation increased the clearance of the pegloticase, decreased its efficacy in lowering serum urate levels, and increased the risk of infusion-related reactions [10]. One study found that infusion-related reactions, such as chest discomfort, flushing, and dyspnea, occurred in approximately 40% of patients [11]. These reactions were resolved by slowing, interrupting, or stopping the infusion. Similar to other biological treatments, physicians began using immunomodulators alongside pegloticase to reduce ADA formation and improve response rate [10]. 

A randomized, placebo-controlled trial found that combining pegloticase with short-term use of MMF significantly reduced serum urate levels over 24 weeks [12]. MMF suppresses T-cell and B-cell proliferation, inhibits lymphocyte recruitment to sites of inflammation, and downregulates adhesion molecule expression, thereby reducing the immune response and the formation of ADA [13]. However, due to its immunosuppressive effects, MMF increases the risk of bacterial, fungal, and viral infections. For example, a retrospective analysis in transplant patients identified four renal transplant patients who developed disseminated VZV infection while on MMF. One patient experienced a primary VZV infection 2.5 months after MMF initiation and three years after transplantation [14]. Another study compared the outcomes of renal transplant recipients using MMF- and non-MMF-based immunosuppression. It was noted that herpes zoster was the most common infection in the MMF group [15]. These findings highlight MMF's potential to increase susceptibility to VZV.

## Conclusions

Advances in gout treatment, such as pegloticase for refractory cases, have improved outcomes. However, its immunogenicity remains a significant challenge, prompting clinicians to use immunomodulators like MMF to mitigate immune-related reactions. While combination therapy has shown promise, the risk of serious infections, such as VZV, underscores the need for careful patient selection and monitoring. This case highlights the serious risks and delicate balance between therapeutic efficacy and safety in managing refractory gout with immunosuppressive regimens.
